# Application of Biomaterials in Diabetic Wound Healing: The Recent Advances and Pathological Aspects

**DOI:** 10.3390/pharmaceutics17101295

**Published:** 2025-10-02

**Authors:** Chenglong Han, Rajeev K. Singla, Chengshi Wang

**Affiliations:** 1 Department of Dermatology, West China Hospital, Sichuan University, Chengdu 610041, China; hancl1997@126.com; 2 Department of Pharmacy and Institutes for Systems Genetics, Center for High Altitude Medicine, Frontiers Science Center for Disease-Related Molecular Network, West China Hospital, Sichuan University, Chengdu 610041, China; 3 School of Pharmaceutical Sciences, Lovely Professional University, Phagwara 144411, Punjab, India; 4 Department of Endocrinology and Metabolism, Laboratory of Diabetes and Metabolism Research, West China Hospital, Sichuan University, Chengdu 610041, China

**Keywords:** diabetic wounds, biomaterials, DNA nanomaterials, peptide hydrogels, extracellular vesicles, cytokines

## Abstract

Diabetic wounds, especially diabetic foot ulcers, pose a major global clinical challenge due to their slow healing and high infection susceptibility. Their typical pathological features include impaired angiogenesis, chronic hypoxia, persistent inflammation, oxidative stress, bacterial colonization, and neuropathy. Traditional treatment methods have limited efficacy, creating an urgent need for innovative therapeutic strategies. In recent years, biomaterials have emerged as a research focus in diabetic wound treatment, owing to their biocompatibility, versatility, and tissue regeneration potential. This article comprehensively reviews the pathological mechanisms of diabetic wounds. It also summarizes the application progress of biomaterials in diabetic wound healing. Over the past decade, researchers have explored the properties, mechanisms of action, and roles of various natural and synthetic biomaterials. These biomaterials include DNA nanomaterials, peptide hydrogels, cells, exosomes, and cytokines. These biomaterials play significant role in promoting angiogenesis, regulating inflammation, inhibiting bacteria, and enhancing cell proliferation and migration.

## 1. Introduction

Diabetes is a complex metabolic disorder affecting the health of hundreds of millions of people worldwide. There are 589 million adults (aged 20–79) with diabetes globally, and this number is projected to rise to 853 million by 2050 [1]. Diabetes leads to hyperglycemia and various complications, including cardiovascular diseases, neuropathy, nephropathy, retinopathy, hearing impairment, dementia, and delayed wound healing [2]. Delayed wound healing is one of the most severe complications associated with diabetes-related impaired wound repair [3]. Severe diabetic wounds can even result in amputation [4]. Studies indicate that 19–34% of diabetic patients develop diabetic foot ulcers, imposing a heavy burden on patients’ quality of life and healthcare systems [5].

**Figure 1:**
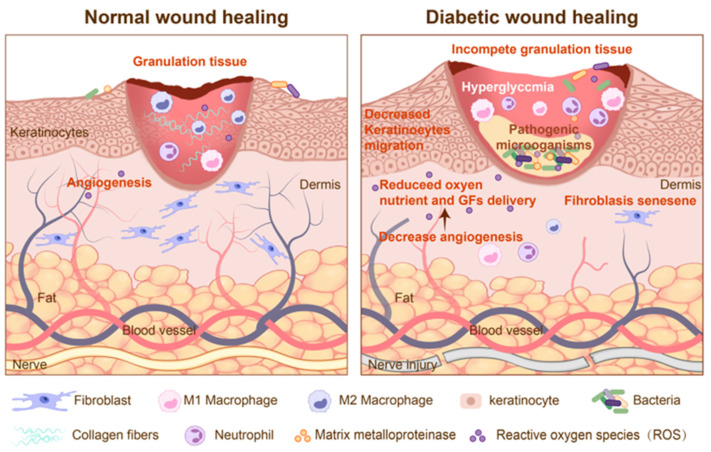
The physiological processes of normal wounds and diabetic wounds.

Wound healing is an orderly biological process involving hemostasis, inflammation, proliferation, and remodeling, with various cell types releasing cytokines and growth factors [6]. During hemostasis, fibrin form clots, and platelets release proinflammatory mediators such as platelet-derived growth factor (PDGF) [7]. In the inflammatory phase, neutrophils and macrophages are recruited to the wound by cytokines, and a temporary extracellular matrix (ECM) is formed [8]. In the proliferative phase, endothelial cells and fibroblasts proliferate and migrate to promote angiogenesis and new ECM formation; meanwhile, matrix metalloproteinases (MMPs) degrade the old ECM, and epithelial cells migrate to initiate re-epithelialization [9]. In the remodeling phase, myofibroblasts restructure the matrix to densify the collagen network, with type I collagen accounting for 80–90% of the total. Enhanced cell apoptosis improves the mechanical resistance of the ECM [10]. Compared to normal wounds, diabetic wounds exhibit dysregulated angiogenesis with chronic hypoxia [11], prolonged inflammatory responses [12], increased oxidative stress [13], persistent bacterial colonization [14], and concurrent neuropathy (Figure 1).

Traditional treatments for diabetic wounds include antibiotic use, surgical debridement, negative pressure suction, oxygen therapy, and dressings such as gauze, bandages, hydrocolloids, and films [15]. These treatments have limitations; for example, conventional dressings and drug therapies may fail to effectively address the complex wound microenvironment. Thus, managing diabetic wounds remains a clinical challenge.

Compared with traditional treatments, biomaterials have become a more promising strategy for diabetic wound therapy due to their unique advantages. DNA nanomaterials can effectively regulate chronic wound inflammation through their inherent anti-inflammatory properties or drug-loading capability. Peptide hydrogels achieve efficient infection control and reduce bacterial resistance risk via inherent antibacterial activity or synergy with antimicrobials. Both cells and extracellular vesicles can promote angiogenesis, accelerate cell proliferation, and inhibit inflammatory responses. Moreover, biomaterials possess multi-functional integration characteristics, enabling simultaneous wound moisturization, anti-inflammation, antibacterial activity, and tissue repair to address multi-dimensional challenges in diabetic wound healing from multiple targets. However, biomaterials still have certain risks and limitations in application: cell therapy faces potential carcinogenicity and cell variability issues; extracellular vesicles are limited by technical bottlenecks such as low isolation and purification efficiency and high quality control difficulty.

This review systematically summarizes the pathological mechanisms of diabetic wound healing, the progress in applying biomaterials for this purpose, and future development directions. By reviewing existing literature, it analyzes the multifunctional roles of biomaterials in promoting wound healing, their potential, and limitations in clinical applications, aiming to provide a theoretical basis and practical guidance for future research.

## 2. Pathological Mechanisms of Diabetic Wound Healing

### 2.1. Angiogenesis Disorders and Ischemic Microenvironment

Angiogenesis is fundamental to wound healing, relying on the proliferation and migration of vascular endothelial cells (ECs) [16]. Hyperglycemia and oxidative stress can impair endothelial function through multiple pathways, leading to endothelial dysfunction.

Chronic hyperglycemia induces non-enzymatic glycosylation of proteins and generating advanced glycation end products (AGEs) [17]. AGEs stimulate increased endothelial cell permeability [18]. The binding of AGEs to the receptor for AGEs (RAGE, a plasma membrane-localized receptor) activates various downstream pathways [19], such as p21ras and mitogen-activated protein kinase (MAPK) pathways, followed by nuclear factor-κB (NF-κB) activation. This upregulates pro-apoptotic proteins (Bax), inducible nitric oxide synthase (iNOS), and tumor necrosis factor-alpha (TNF-α), subsequently increasing caspase-3 activity and ultimately causing EC apoptosis [20]. Hyperglycemia increases diacylglycerol (DAG) levels in cultured ECs in a time-dependent manner, activating protein kinase C (PKC). PKC activation disrupts the endothelial barrier and increases transendothelial albumin permeability. It also upregulates the expression of nicotinamide adenine dinucleotide phosphate (NADPH) oxidase subunits gp91phox and p22phox, leading to p38 MAPK phosphorylation, dephosphorylation of platelet-derived growth factor receptor β (PDGFRβ), impairment of its downstream signaling, and ultimately vascular cell apoptosis [21]. Additionally, forkhead transcription factor (FOXO1) promotes EC senescence and hinders their proliferation by affecting pro-apoptotic proteins (Bim) and cell cycle inhibitors (e.g., p53 and p21).

Endothelial progenitor cells (EPCs) remain a subject of debate [22]. They can proliferate, migrate, adhere, differentiate into endothelial cells, repair damaged endothelial cells, and secrete angiogenic factors (e.g., vascular endothelial growth factor (VEGF)), playing a crucial role in angiogenesis in ischemic tissues [23]. Hyperglycemia and oxidative stress affect the quantity and function of EPCs [24]. Current studies show that AGEs/RAGE binding downregulates Akt and Cox-2, impairing late EPC function [25]. Reactive oxygen species (ROS) inhibit Akt phosphorylation and endothelial NOS (eNOS) expression, reduce nitric oxide (NO) activity, suppress the PI3K/Akt/eNOS signaling pathway, induce EPC apoptosis, and cause defects in EPC migration as well as lumen formation [26]. H_2_O_2_ induces EPC apoptosis through a FoxO3a-dependent mechanism [27]. Activation of the Akt/p53/p21 signaling pathway induced by oxidized low-density lipoprotein (ox-LDL) is positively correlated with accelerated senescence of EPCs in diabetic patients [28].

Nitric oxide is the most important bioactive substance produced by endothelial cells, and impaired NO bioavailability is a hallmark of endothelial dysfunction [29]. Hyperglycemia and oxidative stress reduce NO bioavailability through multiple mechanisms. AGEs decrease NO production and eNOS expression. Moreover, hyperglycemia-induced PKC activation promotes eNOS gene expression and triggers eNOS uncoupling, thereby reducing NO bioavailability. In diabetic wounds, reduced tetrahydrobiopterin levels cause NOS dissociation into monomers, generating superoxide radicals that oxidize tetrahydrobiopterin and decreasing NO production [30]. Oxidative stress activates FOXO1, increasing inducible NOS transcription and interfering with eNOS function [21].

Various growth factors are critical for promoting angiogenesis, such as VEGF [31]. The complex pathological state of diabetic wounds reduces the quantity and activity of growth factors. ROS decrease the transcriptional activity of VEGF, angiopoietin 2, and fibroblast growth factors as well as inhibiting angiogenesis [32]. Hyperglycemia impairs macrophage function and reduces the migration and proliferation of keratinocytes and fibroblasts, leading to insufficient production of VEGF and other pro-angiogenic mediators, thus impairing angiogenesis [26].

In diabetic wounds, the Ang1/Ang2/Tie2 complex is a vascular maturation pathway associated with angiogenesis defects [33]. A reduced Ang1/Ang2 ratio impairs the ability of diabetic wound vessels to mature.

MicroRNAs (miRNAs) are another class of molecules that regulate angiogenesis and wound repair [34]. Specific miRNAs have been shown to have altered expression in diabetic wound healing [35]. miR-126 overexpression enhances the pro-angiogenic capacity of progenitor cells and reduces tissue factor expression, but its levels are decreased in type 2 diabetic patients [36]. miR-503 is upregulated in the serum of diabetic patients, inhibiting pericyte VEGF expression and increasing vascular permeability [37].

Insufficient angiogenesis leads to local oxygen deprivation and a high oxygen demand of wound cells, forming an ischemic-hypoxic microenvironment that triggers a series of pathological changes [38]. Severe hypoxia induces cell apoptosis, with ROS playing an important role [39]. Hypoxia-inducible factor 1-alpha (HIF-1α) is a key regulator of oxygen homeostasis, promoting angiogenesis by activating the transcription of various angiogenic factors, including VEGF and fibroblast growth factor-2 (FGF-2) [40]. HIF-1α inhibition, along with exacerbated hypoxia and impaired cellular responses to hypoxia, is a key pathogenic factor for delayed diabetic wound healing. Hyperglycemia destabilizes HIF-1α expression through VHL-dependent and MGO-mediated mechanisms [41]. Additionally, hyperglycemia and high ROS levels inhibit HIF-1α activity [42]; however, these mechanisms require further validation.

Hypoxia also affects the normal function of key cellular participants in healing, including fibroblasts and keratinocytes [43]. For example, fibroblasts exhibit reduced function under prolonged hypoxia, leading to insufficient ECM deposition and impaired wound closure [44]. Furthermore, hypoxia promotes the recruitment of inflammatory cells to the wound, exacerbating inflammation and further hindering healing process [45].

### 2.2. Chronic Inflammation and Immune Dysfunction

Prolonged inflammation in diabetic wounds is a key factor in delayed healing. Immune cells, particularly macrophages, play a crucial role in diabetic wounds [46]. In the acute phase of healing, increased interleukin (IL)-10 expression in diabetic wounds reduces toll-like receptor (TLR) signaling and pro-inflammatory cytokine production, delaying macrophage recruitment and impairing healing [47]. Polarized macrophages in wounds are classified as pro-inflammatory (M1) or anti-inflammatory/wound healing (M2) [48]. M1 macrophages appear after injury, clear bacteria, foreign debris, and dead cells through phagocytosis, and produce pro-inflammatory cytokines such as IL-1, IL-6, TNF-α, and interferon (IFN)-γ [49]. M2 macrophages have anti-inflammatory and immunomodulatory effects, are polarized by Th2 cytokines (e.g., IL-4 and IL-13), and produce anti-inflammatory cytokines such as IL-10 and TGF-β [50]. M2 macrophages have four subtypes [51]: M2a which promotes cell proliferation and ECM production [52], M2b which inhibits inflammation [53], M2c has matrix remodeling capacity [54], while M2d has angiogenic and remodeling activity [55]. Diabetic tissues show increased macrophage numbers [47]. In a high-glucose environment, macrophages exhibit an M1 inflammatory phenotype [56], possibly related to oxidative stress and ROS production under hyperglycemia [57]. Cytokines and chemokines produced by M1 macrophages, such as IL-1β, IFN-γ, TNF-α, and IL-12, induce local and systemic inflammation [47].

IL-1β is a key inflammatory cytokine, with its active form generated through activation of the nucleotide-binding and oligomerization domain (NOD)-like receptor pyrin domain-containing 3 (NLRP3) inflammasome [58]. This activation is inhibited by peroxisome proliferator-activated receptor-γ (PPAR-γ) [59]. In diabetic patients, reduced PPAR-γ levels enhance NLRP3 inflammasome activation, leading to uncontrolled IL-1β secretion. Excessive active IL-1β perpetuates a positive feedback loop maintaining inflammation in diabetic wounds [60].

M1 macrophages in wounds secrete large amounts of proteases, including MMPs [61]. MMPs degrade collagen and other ECM components, playing an important role in the remodeling phase of wound healing [62]. The balance between MMPs and their endogenous tissue inhibitors (TIMPs) is critical for wound healing [63]. Overexpressed pro-inflammatory cytokines in chronically hyperglycemic wounds upregulate MMP synthesis and downregulate TIMP expression, disrupting the MMP/TIMP ratio, degrading ECM, and delaying wound healing [64]. Degraded ECM also alters immune cell activity by shaping their activation, differentiation, and survival [65], attracting more macrophages, increasing inflammation, and maintaining wound chronicity.

The pathological state of diabetes affects macrophage function [66]. In a diabetic environment, M1 macrophage phagocytosis is impaired, possibly contributing to the susceptibility of diabetic patients to infections [67]. The AGE/RAGE signaling axis in diabetic patients impairs M1 macrophage phagocytic function [68]. Additionally, diabetes affects the ability of macrophages to clear apoptotic cells [69]. In diabetic wounds, M1 macrophages fail to phagocytose apoptotic neutrophils [70]. Excessive neutrophils not phagocytosed by wound macrophages produce neutrophil extracellular traps (NETs), which activate the NLRP3 inflammasome through TLR-4/TLR-9/NF-κB and ROS/TXNIP signaling pathways, further amplifying inflammation and oxidative stress [54].

Phagocytosis of neutrophils by M1 macrophages may be a key driver of their conversion to the M2 phenotype [71]. Wound infection, high glucose, and AGEs alter macrophage phenotype [72]. Impaired M1-to-M2 macrophage polarization is a major cause of chronic inflammation and impaired wound healing in diabetic patients [33]. Impaired M2 polarization of macrophages in diabetic wounds reduces their anti-inflammatory capacity and lowers levels of pro-healing factors such as IGF-1, TGF-β1, VEGF, and IL-10, exacerbating chronic inflammation and delaying wound healing [60].

Neutrophils also contribute to delayed diabetic wound healing. In early diabetic wounds, neutrophil migration is impaired. Hyperglycemia inhibits formyl peptide receptor (FPR)-mediated chemotaxis in neutrophils, delaying their recruitment and reducing bactericidal capacity [73]. In late-stage diabetic wounds, neutrophil extracellular trap formation (NETosis) increases, which may be associated with delayed healing [74]. NETs produced by neutrophils are important for killing microorganisms but can directly induce epithelial and endothelial damage and promote systemic inflammation [75]. Neutrophil elastase derived from NETs is elevated in diabetic wounds, degrading the wound matrix and impairing healing [76]. Studies show that peptidylarginine deiminase 4 (PAD4)-mediated histone citrullination is enhanced in diabetic wounds, with excessive NET formation [77] associated with NLRP3 inflammasome activation [78]. Milk fat globule-epidermal growth factor VIII (MFG-E8) is critical for regulating NLRP3-mediated NETosis, but glycosylated MFG-E8 in a high-glucose environment fails to effectively control NETosis [79]. In diabetes and hyperglycemia, neutrophils switch their energy source from glycolysis and the pentose phosphate pathway to the polyol pathway; polyol pathway activation consumes NADPH, causing abnormal NET function [80]. Additionally, neutrophil function is impaired in diabetic wounds, their ability to upregulate ROS at infected wound sites is compromised, thus reducing bactericidal activity [81]. Impaired glucose metabolism in neutrophils under diabetic conditions reduces their energy, impairing phagocytosis [82]. Exposure to AGEs reduces neutrophil viability and accelerates apoptosis [83].

Other immune cells also exhibit abnormalities in diabetic wounds. Studies show that diabetic patients have reduced naive T cell numbers, poor effector T cell proliferation, and limited T cell receptor (TCR) diversity [84]. In a diabetic environment, reduced IL-15 production and impaired mTOR activation alter the homeostasis of dendritic epidermal T cells (DETCs) in diabetic mice, lowering IGF-1 levels and impairing wound healing [85]. Under hyperglycemia, myeloid-derived suppressor cells (MDSCs) abnormally proliferate and tend to differentiate into M1 macrophages [86]. Mast cells (MCs) and MC-derived mediators participate in all wound healing stages; increased degranulation of skin MCs in diabetes is associated with impaired wound healing [87]. In diabetic wounds, dendritic cells (DCs) participate in clearing apoptotic cells, and inhibiting SLC7A11 function accelerates wound healing in diabetic mice [88].

Multiple inflammatory pathways are persistently activated in diabetic wounds. NF-κB is a key molecule in inflammatory responses, inducing the expression of various inflammatory factors and adhesion molecules, and amplifying the inflammatory cascade [89]. In diabetes, hyperglycemia and ROS activate the NF-κB signaling pathway, promoting M1 polarization of phagocytes and pro-inflammatory cytokine transcription [90]. Inflammatory factors further increase ROS production by activating nicotinamide adenine dinucleotide phosphate oxidase (NOX) enzymes, forming a vicious cycle [91]. The AGEs-RAGE axis participates in abnormal inflammatory responses in diabetic wounds through multiple mechanisms; for example, AGEs accumulated in the diabetic wound environment affect macrophage functional status through RAGE, contributing to the imbalance between pro-inflammatory and repair processes [92]. Epigenetic regulation of gene expression in diabetes also affects immune cell phenotypes and inflammatory responses. The COX-2/PGE_2_ pathway is abnormally expressed in macrophages of diabetic wounds, increasing levels of pro-inflammatory cytokines such as IL-1β and IL-12 [93]. Histone deacetylase 6 (HDAC6) affects inflammation by regulating microtubule dynamics and cytokine secretion [94]. Immune cell-derived miRNAs, such as miR-497, miR-132, and miR-21, influence wound healing by regulating inflammatory factors and macrophage polarization [95].

### 2.3. Bacterial Infection and Biofilm Formation

Multiple mechanisms contribute to the susceptibility of diabetic patients to infections. Hyperglycemia provides excess nutrients for microorganisms. In a diabetic environment, neutrophil chemotaxis, phagocytosis, and microbial killing functions are impaired, reducing innate immunity [96]. The hypoxic microenvironment due to poor angiogenesis promotes anaerobic bacterial colonization [97]. Diabetic wounds show downregulated expression of antimicrobial peptides (AMPs), particularly cathelicidin (LL-37), reducing antibacterial and wound repair capacities [98]. Consequently, microbial flora (mainly bacteria) colonize wounds through various inducing factors [99]. Bacterial virulence factors, especially bacterial proteases (serine, cysteine, and metalloproteases), play important roles in the pathogenesis of delayed diabetic wound healing [100].

Diabetic wound infections are characterized by high bacterial diversity, mainly skin commensals, including opportunistic pathogens and anaerobes [101]. Common pathogenic bacteria include *Staphylococcus aureus* (30–40%), *Pseudomonas aeruginosa* (15–20%), and *Escherichia coli* [102]. In diabetic wounds, highly complex microbial communities form biofilms through multiple steps, including random settlement of early bacterial colonizers, interspecies competition, and niche differentiation [103]. Biofilms are enclosed microbial communities embedded in a mucus-like extracellular polymeric matrix, with extracellular polymers (EPS, containing polysaccharides, proteins, and nucleic acids) surrounding microbial cells. EPS accounts for 80–85% of the volume and microorganisms only 15–20% [104,105]. The prevalence of biofilms in chronic wounds is 78.2% [106].

Extracellular polymers can adhere to biological or non-biological surfaces and act as a physical protective barrier against host immune defenses and antimicrobial therapies [107]. Bacteria in biofilms resist host immune defenses through multiple mechanisms: (1) limited penetration of immune cells and their products into biofilms [108]; (2) reduced phagocytosis of biofilm bacteria by host immune cells [109]; (3) quorum sensing (QS) in biofilm bacteria [110]; and (4) genetic control of biofilm bacterial resistance [111]. In fact, persistent immune activation and infection cause collateral damage to surrounding tissues and chronic inflammation, impairing diabetic wound healing [112].

Another important feature of biofilms is antimicrobial resistance. Antimicrobial tolerance arises from multiple mechanisms: (1) the physical protective barrier function of EPS limits antimicrobial penetration into biofilms; (2) biofilms promote the formation of persister cells resistant to antimicrobial eradication [113]; (3) QS activation in biofilm bacteria upregulates the expression of virulence genes, including antimicrobial resistance genes [114]; (4) increased horizontal gene transfer (HGT) rates among biofilm community members spread antibiotic resistance traits [115]; and (5) natural selection of resistant strains by antibiotics, especially broad-spectrum ones, and antibiotic abuse.

The microbial profile of diabetic wound infections exhibits significant spatiotemporal heterogeneity, but the high detection rate of drug-resistant bacteria is a global issue. A meta-analysis integrating data from 23 studies found that the methicillin-resistant *S. aureus* (MRSA) colonization rate in diabetic patients is 9.20% (95% CI: 6.26–12.63%), and in diabetic foot ulcer (DFU) patients, the MRSA infection rate reaches 16.78% (95% CI: 13.21–20.68%) [116]. Regional differences are prominent in drug-resistant bacteria distribution: Gram-positive bacteria (GPB) predominate in diabetic foot infection (DFI) in developed countries from North America and Europe, with MRSA detection rates of 12–20% in DFI, while Gram-negative bacteria (GNB) are dominant in developing countries from Asia and Africa [117]. Notably, drug-resistant bacteria detection rates show an upward trend over time. Systematic review data indicate that between 2001 and 2020, the MRSA isolation rate in Chinese DFI patients increased from 8.24% to 11.56%, and the *Pseudomonas aeruginosa* infection rate from 10.14% to 11.72%. This temporal trend is closely related to the spread of resistance genes under antibiotic selective pressure, suggesting that drug resistance is worsening with the rising incidence of diabetes. Antibiotic resistance traps clinical treatment of diabetic wounds in a vicious cycle of reduced efficacy and increased recurrence rates, imposing dual pressures on patient prognosis and healthcare systems.

### 2.4. Oxidative Stress and Cellular Damage

In diabetes, oxidative stress is involved in its pathogenesis, leading to insulin resistance, dyslipidemia, β-cell dysfunction, and impaired glucose tolerance. On the other hand, hyperglycemia-mediated oxidative stress causes inflammation and cellular/tissue damage through lipid peroxidation, DNA damage, and mitochondrial dysfunction, increasing the risk of hypertension, cardiovascular diseases (including myocardial infarction, atherosclerosis, and stroke), and other serious health issues [118]. Oxidative stress largely results from increased ROS production and decreased activity of antioxidant defense systems [119].

Hyperglycemia increases ROS through multiple mechanisms, leading to vascular complications [118] (Paragraphs 2–4 of Section 2.1 above). Current research suggests that glucose-mediated vascular damage involves four main molecular mechanisms: increased polyol pathway flux, increased hexosamine pathway flux, increased AGE formation, and activation of protein kinase C (PKC) isoforms [118,120].

The polyol pathway, also known as the sorbitol-aldose reductase pathway, reduces glucose to sorbitol, which is then oxidized to fructose [121]. Under hyperglycemia, when glucose exceeds the capacity of glycolysis, hexokinase (HK) becomes saturated, and excess glucose enters the polyol pathway, where aldose reductase (AR) reduces it to sorbitol. This reaction oxidizes NADPH to NADP. Sorbitol dehydrogenase (SDH) can then oxidize sorbitol to fructose, generating NADH (nicotinamide adenine dinucleotide) from its oxidized form NAD. NADPH is an important cofactor for preserving and regenerating the antioxidant reduced glutathione (GSH). Intracellular NADPH levels decrease in the polyol pathway. Additionally, AR and glutathione reductase (GSR) compete for the NADPH cofactor, further reducing intracellular GSH and impairing cellular antioxidant capacity. Moreover, fructose produced by the polyol pathway can further react to form 3-deoxyglucose and 3-deoxyglucosone, which can glycate proteins and lead to AGE production [122].

The hexosamine pathway is part of glycolysis [123]. Under hyperglycemia and under the catalysis of glutamine: fructose-6-phosphate amidotransferase (GFAT), it converts the glycolytic intermediate fructose-6-phosphate to UDP-N-acetylglucosamine (UDP-GlcNAc). Subsequent nucleocytoplasmic protein glycosylation often leads to pathological changes in gene expression and promoting diabetic complications [124].

Advanced glycation end products form through non-enzymatic reactions between free amino groups of proteins and carbonyl groups of reducing sugars or other carbonyl compounds [124]. Glucose (or other reducing sugars, such as fructose) reacts with reactive amino groups to form reversible Schiff bases, which then rearrange into more stable Amadori products. In late glycosylation, early glycosylation products form irreversible AGEs [125]. Glycosylation of proteins and lipoproteins interferes with their normal functions by disrupting molecular conformation, altering enzyme activity, reducing degradability, and interfering with receptor recognition [126]. AGEs interact with the specific cell surface receptor RAGE, altering intracellular signaling, gene expression, release of pro-inflammatory molecules, and free radical production [127]. In a long-term hyperglycemic environment, AGEs are actively produced and accumulate in blood and various tissues, interacting with RAGE to cause vascular complications of diabetes through various mechanisms [19] (Paragraphs 2–4 of Section 2.1 above).

Protein kinase C is a multigene family of related serine/threonine kinases, including at least 11 isoforms, most triggered by the lipid second messenger diacylglycerol (DAG) [128]. Hyperglycemia can chronically activate the DAG-PKC pathway in the vasculature. PKC activation has multiple pathogenic outcomes, playing a role in diabetes-related vascular diseases [129] (Paragraphs 2–4 of Section 2.1 above).

The four main pathways involved in the pathogenesis of diabetic complications are activated by a common upstream event: excessive production of ROS (like superoxide) caused by an intracellular environment of high glucose [130]. In diabetic cells with high intracellular glucose, more glucose is oxidized in the tricarboxylic acid (TCA) cycle, pushing more electron donors (NADH and FADH2) into the electron transport chain. Consequently, the voltage gradient across the mitochondrial membrane increases until a critical threshold is reached, at which point mitochondrial electron transport chain blockage leads to a single electron transfer to molecular oxygen, generating superoxide [131]. Hyperglycemia-induced superoxide reduces the activity of the glycolytic enzyme, glyceraldehyde-3-phosphate dehydrogenase (GAPDH), by modifying the enzyme with ADP-ribose polymers, increasing levels of all glycolytic intermediates upstream of GAPD. Elevated levels of the upstream glycolytic metabolite glyceraldehyde-3-phosphate activate the AGE formation pathway and the classical PKC pathway [132]. Increased amounts of the upstream glycolytic metabolite fructose-6-phosphate enhance flux through the hexosamine pathway [133]. Finally, GAPDH inhibition increases intracellular glucose, enhancing flux through the polyol pathway [134]. The specific inhibitor poly (ADP-ribose) polymerase (PARP) prevents ADP-ribose modification and hyperglycemia-induced reduction in GAPDH activity [135]. However, hyperglycemia causes excessive production of ROS and DNA single-strand breaks, stimulating PARP and leading to changes in GAPDH and thus reduced activity [136].

Cellular antioxidant defense mechanisms include enzymatic and non-enzymatic strategies [137]. Common antioxidants include enzymes such as superoxide dismutase (SOD), catalase, glutathione peroxidase, and GSR, as well as vitamins A, C, and E, and GSH [138]. In diabetes, hyperglycemia and ROS impair antioxidant enzyme activity, possibly related to spontaneous glycosylation of key antioxidant enzymes (e.g., catalase, SOD, glutathione-S-transferase, glutathione peroxidase, and GSR) [139]. Glycosylation also inactivates antioxidant enzymes; for example, fluxes of nitric oxide and superoxide inactivate and nitrosylate human SOD. On the other hand, high oxidative stress in diabetes consumes large amounts of GSH, vitamin C, and vitamin E, reducing tissue concentrations of low-molecular-weight antioxidants (e.g., GSH and vitamin E). Additionally, diabetic patients often have nutritional metabolic disorders, reducing intake of exogenous antioxidants such as vitamin E and vitamin C [140]. These factors collectively lead to insufficient cellular antioxidant capacity in diabetes.

Persistent oxidative stress promotes senescence of fibroblasts, endothelial cells, and keratinocytes, affecting the formation of granulation tissue, blood vessels, and epithelial cells, thereby delaying wound healing [137].

### 2.5. Neuropathy

Neuropathy also impairs the healing process of diabetic wounds, with its mechanisms acting throughout wound formation, progression, and repair. Peripheral sensory nerve damage in diabetes causes loss of protective sensation in the feet, making patients unable to detect minor trauma (e.g., slight abrasions and low-temperature burns) and prone to occult wounds. Autonomic nerve damage inhibits foot sweat gland secretion, leading to dry, cracked skin, impaired skin barrier function, disrupted vasomotor regulation, reduced skin resistance, and higher risk of secondary wound infection.

Furthermore, neuropeptides released by autonomic nerves regulate the expression and function of multiple diabetes-related dysregulated cytokines, including IL-1, IL-6, IL-8, IL-10, and TNF-α [141].

Moreover, neuropathy affects sympathetic and parasympathetic nerves innervating blood vessels, resulting in vasomotor dysfunction. Vascular dysfunction further exacerbates local tissue ischemia-hypoxia, which in turn worsens nerve damage, forming a “neuropathy → vascular disorder → ischemia-hypoxia → aggravated nerve damage” vicious cycle and further delaying wound healing [142].

## 3. Design and Application of Biomaterials in Diabetic Wounds

Biomaterials have received extensive attention in diabetic wound treatment due to their biocompatibility, customizable therapeutic targets, and superior sustained delivery capabilities. Various natural and synthetic biomaterials (such as DNA nanomaterials, peptide hydrogels, cells, exosomes, and cytokines) can promote angiogenesis, regulate inflammatory responses, exert antibacterial effects, and enhance cell proliferation and migration by delivering active and inactive substances through carrier platforms, ultimately promoting tissue repair. This section focuses on the application of immunomodulatory hydrogels in diabetic wound healing.

### 3.1. DNA Nanomaterials

With the in-depth development of nanotechnology and engineering, biomolecule-functionalized nanomaterials, especially DNA nanomaterials, are showing vigorous vitality. As novel biomaterials, DNA nanostructures, with their unique advantages of structural programmability, biocompatibility, biodegradability, high specificity, and modifiability, have initiated diverse application explorations in biomedicine. After decades of development, DNA nanostructures have evolved into various configurations, such as 3D tetrahedral DNA nanostructures (TDNs) and DNA origami [143]. Currently, the DNA nanostructures explored and applied in diabetic wounds with significant effects are mainly TDNs [144] and DNA hydrogels [145] (Table 1).

#### 3.1.1. Tetrahedral DNA Nanostructures

**Figure 2:**
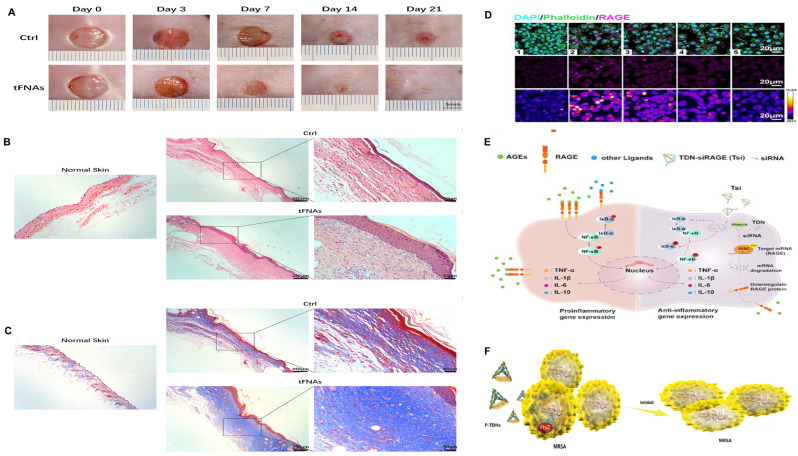
DNA nanomaterials exhibit pro-healing, anti-inflammatory, and antibacterial effects. (**A**): Photographs of skin wounds in diabetic mice treated with either saline or 250 nM tFNAs at different time points. (**B**): H&E staining of wound tissues from the control and tFNA-treated groups on postoperative day 21. Scale bars are 250 or 50 μm. (**C**): Masson’s trichrome staining of wound tissues from the control and tFNA-treated groups on postoperative day 21. Scale bars are 250 or 50 μm. Adapted with permission from [147]. © 2022 The Authors. Cell Proliferation published by Beijing Institute for Stem Cell and Regenerative Medicine and John Wiley & Sons Ltd. (**D**): Immunofluorescence images showing RAGE expression in macrophages after different treatments. (**E**): Schematic illustration of Tsi targeting the RAGE/NF-κB axis in macrophages. Adapted with permission from [152]. Copyright ©2023 American Chemical Society. (**F**): Schematic illustration of MRSA treatment using P-TDNs. Adapted with permission from [148]. Copyright ©2018 American Chemical Society.

Tetrahedral DNA nanostructures, also known as “tetrahedral framework nucleic acids” (tFNAs), are 3D nanostructures self-assembled from four single-stranded DNAs, with a tetrahedral geometry [157]. Compared to natural DNA, TDNs have biocompatibility, programmability, higher density, and better structural stability due to their structural features [158]. In diabetic wound treatment, TDNs can promote cell proliferation and migration, inhibit cell apoptosis, suppress inflammation and oxidative stress, and exert anti-infective effects [159] (Figure 2).

In 2020, Lin et al. found that TDNs increase endothelial cell proliferation and migration and improve angiogenic capacity. Furthermore, this study also found that TDNs inhibit inflammation and oxidative stress by upregulating the expression of antioxidants and downregulating the expression of inflammatory mediators [146]. A 2022 study by Wang on TDNs also supported these findings [147].

Due to their excellent cellular uptake efficiency, good biocompatibility, biodegradability, excellent structural stability, and modifiability, TDNs can achieve precise drug encapsulation and ordered release [160], emerging as a powerful platform for developing next-generation nanomedicines with broad prospects as drug delivery systems in diabetic wound treatment.

Infection is a key factor causing delayed diabetic wound healing. TDNs, as multifunctional delivery carriers for various antimicrobials, enhance their antibacterial efficacy [148]. In 2020, Sun et al. designed ampicillin-loaded TDNs [149]. The study showed that TDNs exert enhanced anti-infective activity by increasing the sensitivity of MRSA cells to ampicillin and downregulating the gene expression of MRSA cells.

TDNs can also deliver antimicrobial peptides (AMPs). In 2020, Liu et al. first combined TDNs with the antimicrobial peptide GL13K to form t-GL13K [150]. The study showed that t-GL13K enhances efficacy against *Escherichia coli* by increasing bacterial uptake of t-GL13K and enhancing deformation of microbial membranes. The combination of microRNA and tFNA also has anti-infective effects. Zhang et al. designed a TDN-based delivery system for antisense oligonucleotides (ASOs), which promotes chronic wound healing by targeting multiple genes to control infection [161].

TDNs can deliver small-molecule drugs with other functions. In 2022, Lin et al. designed a REGRT healing peptide delivery system, p@tFNAs, based on tetrahedral framework nucleic acids (TDNs) [151]. Their study showed that p@tFNAs enhance angiogenic capacity in a high-AGE environment and have antioxidant functions. In vivo experiments demonstrated that p@tFNAs promote epidermal coverage, angiogenesis, and collagen deposition. In 2023, Cai et al. designed a TDN delivery system conjugated with siRNA [152]. This system facilitates Tsi penetration into cell membranes, thereby targeting and silencing the RAGE gene, blocking activation of harmful downstream signaling pathways, and inhibiting AGE-induced inflammatory responses.

Despite encouraging results with TDN-based therapies, obstacles remain there, hindering translational potential. Particularly, as TDNs are loaded with increasingly complex cargoes, their cellular uptake capacity decreases, weakening their delivery function [162]. To address this, in 2024, Ge et al. designed a tFNA-Apt02-DMOG complex (TACD) structure to improve delivery function [153]. They integrated Apt02 (a DNA aptamer mimicking VEGF-A activity) and dimethyloxalylglycine (DMOG) small molecules into the TACD structure through template-based click chemistry and toehold-mediated strand displacement reactions. Studies have shown that TACD accelerates the healing of skin defects in diabetic mice by promoting re-epithelialization, angiogenesis, and collagen deposition.

#### 3.1.2. DNA Hydrogels

DNA hydrogels are another type of DNA biomaterial widely used in diabetic wounds. They not only share characteristics with other hydrogels but also exhibit biocompatibility, biodegradability, programmability, and stimuli responsiveness [163]. DNA hydrogels are mainly classified into pure DNA hydrogels and hybrid DNA hydrogels. In pure DNA hydrogels, the network is exclusively constituted by interwoven DNA strands. In contrast, hybrid DNA hydrogels utilize a synthetic polymer as the primary scaffold, where DNA nanostructures function either as dynamic cross-links or as embedded therapeutic agents [164]. DNA hydrogels are formed through chemical and physical cross-linking. Chemical cross-linking involves covalent interactions within linear DNA strands or between DNA and other polymers, requiring chemical linkers and forming permanent and irreversible bonds [165]. Physical cross-linking relies on non-covalent intermolecular interactions such as hydrogen bonds, metal-DNA coordination, and electrostatic interactions, without involvement of chemical linkers [166]. Thus, chemically cross-linked hydrogels show stronger physiological and structural stability while physically cross-linked hydrogels exhibit better biocompatibility, biodegradability, and minimal cytotoxicity.

DNA hydrogels play three main roles in diabetic wounds: (1) significant antioxidant function based on the natural ability of DNA strands; (2) excellent and stable delivery platforms for drug loading and release [167]; and (3) therapeutic drugs targeting genes to regulate biological processes at the genetic level [168].

The biocompatibility, binding ability, and active targeting capability of DNA hydrogels make them promising carriers for drug delivery systems, capable of loading various inorganic NPs, small-molecule drugs, and functional biomacromolecules [169].

In 2022, Wang et al. designed a physically cross-linked DNA hydrogel encapsulating IL-33 [170]. They found that the porous microstructure and biodegradable properties of the IL-33-loaded hydrogel promote long-term sustained release of IL-33 in vitro and in vivo. The DNA hydrogel itself exhibits antioxidant properties without negative effects on cell viability. The study showed that the multifunctional IL-33-loaded hydrogel promotes re-epithelialization, endothelial cell proliferation, angiogenesis, and ECM formation, while promoting M1-to-M2 macrophage polarization through the IL-33-ILC2 axis, downregulating inflammatory factor expression, and accelerating diabetic wound healing. In 2023, Li et al. developed multifunctional AgNCs-hydrogels, combining DNA hydrogels with silver nanoclusters (AgNCs), which have enhanced antibacterial properties and ROS-scavenging ability, promoting diabetic wound healing [171]. In another study, Obuobi et al. developed DNA hydrogels constructed using interactions between the highly polyanionic backbone of DNA nanostructures and cationic AMPs, for delivering AMPs in wound treatment [154]. The study showed that the formulated L12-loaded DNA hydrogel exhibits significant efficacy against *S. aureus* infections.

In hybrid DNA hydrogels, DNA nanostructures, including plasmid DNA, DNA fragments, and DNA aptamers, act as therapeutic drugs to regulate biological processes at the genetic level, promoting diabetic wound healing.

In 2023, Hwang et al. developed a gene-activated hyaluronic acid collagen matrix (GAHCM), which retains plasmid DNA multimers encoding growth factors in a collagen-based hydrogel through chain invasion of collagen-mimetic peptides (CMPs) with collagen [155]. They found that CMP modification enhances VEGF gene expression, increasing VEGF-A activity, ultimately facilitating granulation tissue formation, vascular maturation, re-epithelialization, and improved wound healing. Polydeoxyribonucleotides (PDRN), first extracted from human placenta, are DNA fragments containing 50–2000 bases [172]. They initiate intracellular signaling through A2 purinergic receptors, influencing tissue regeneration and anti-inflammation, thereby promoting wound healing. In 2018, Li et al. developed a PDRN-loaded sodium alginate (SA)/short-chain chitosan (SCS) gel (PEI/PDRN@SA/SCS gel), which promotes cell proliferation and wound healing through slow PDRN release [156].

Although DNA hydrogels are generally considered biocompatible, some studies suggest that DNA degradation products may trigger inflammatory responses or interfere with the host immune system. The lack of long-term toxicity and immunogenicity studies raises concerns about their safety.

### 3.2. Peptide Hydrogels

Hydrogels are novel materials with a 3D network structure, featuring high water content, adjustable mechanical stability, and biocompatibility. Compared to ordinary polymer hydrogels, peptide hydrogels exhibit superior biocompatibility and biodegradability, and they can mimic the ECM and exert anti-infective effects [173]. Thus, peptide hydrogels have been widely applied in multiple stages of diabetic wound healing, including infection control, inflammation regulation, cell proliferation, and angiogenesis (Table 2).

Anti-infection is perhaps the most common application of peptide hydrogels in diabetic wound healing, mainly utilizing the self-assembling structure of peptide-based materials, bactericide-carrying capacity, and antimicrobial peptides [180].

Neutral peptides can self-assemble into nanofibers, which can disrupt bacterial cell membranes. The simplest neutral self-assembling AMP is diphenylalanine nanoassembled peptide (FF) [181]. FF affects the permeability and depolarization of bacterial outer and inner membranes, thereby exhibiting antibacterial activity [182]. In 2023, Sharma et al. synthesized an Fmoc-phenylalanine nanofiber hydrogel [174]. Their study showed that Fmoc-phenylalanine nanofibers can disrupt bacterial cell walls, exhibit antibacterial activity against both Gram-positive and Gram-negative bacteria, and serve as effective alternatives to conventional antibiotics.

Peptide-based hydrogels can be combined with bactericides to control or prevent infections, thus emerging as an effective approach for treating diabetic wounds [183]. Silver nanoparticles (AgNPs) are common antimicrobials with broad antibacterial activity against various Gram-positive and Gram-negative bacteria [184]. Peptide-based hydrogels can carry AgNPs to exert antibacterial effects. In 2020, D’Souza et al. designed an L9-Ag hydrogel, which inhibits the growth of Gram-negative *E. coli* and *S. aureus* without cytotoxicity to 3T3 fibroblasts [175].

Antimicrobial peptides are a group of widely distributed important antibacterial substances with broad antibacterial activity. They destabilize lipid bilayers through electrostatic interactions with anionic components of bacterial membranes, exerting antibacterial effects. However, they are easily degraded by enzymes in organisms and may have cytotoxicity. Studies have shown that AMPs covalently immobilized on materials such as hydrogels retain their antibacterial activity while avoiding cytotoxicity [185]. In 2021, Atefyekta et al. developed an antimicrobial hydrogel by covalently immobilizing cationic AMP (RRP9W4N) on ordered amphiphilic mesoporous hydrogels, which exerts highly effective contact-killing effects against a wide range of bacterial species, including MRSA and multi-drug resistant (MDR) *E. coli* [176].

Finely designed peptide hydrogels also have functions in inflammation regulation, promotion of cell proliferation, and angiogenesis. In 2025, Lu et al. constructed a polypeptide complex hydrogel by loading Ac2-26 (Ac) peptide into a mixture of hyaluronic acid (HA) and β-cyclodextrin (β-CD) with the addition of biocompatible polyvinyl alcohol (PVA) and polyethylene glycol (PEG) [177]. The study found that this hydrogel ultimately promotes diabetic wound healing by regulating macrophage polarization, facilitating collagen deposition, and enhancing neovascularization.

Sanapalli et al. developed a hydrogel where human β-defensin-2 (HBD-2)-loaded poly (lactic-co-glycolic acid) (PLGA) nanoparticles were impregnated into a collagen/chitosan (COL-CS) composite scaffold [178]. Studies showed that this hydrogel could downregulate the expression of MMP-9 and inflammatory mediators, accelerate cell migration and angiogenesis, and promote wound healing. Additionally, this hydrogel exhibited excellent antibacterial activity. Tian et al. developed a gelatin nanofiber composite hydrogel (designated as PGF@ALG/PLGA hydrogel) functionalized with branched polyethyleneimine (PEI). This hydrogel exhibits favorable biocompatibility and can provide a biomimetic microenvironment for potential tissue regeneration. Studies have demonstrated that it not only exerts significant antibacterial efficacy but also promotes the polarization of macrophages toward the M2 phenotype, thereby synergistically accelerating the transition from the inflammatory phase to tissue regeneration [179].

Additionally, peptide hydrogels can serve as drug delivery platforms, targeting and delivering related drugs and biological structures to promote diabetic wound healing [186]. In 2022, Kim et al. designed a self-assembling peptide hydrogel (SAPH) loaded with the active VEGF epitope domain SLan, which improves wound healing in diabetic rat models by promoting new blood vessel deposition and re-epithelialization in the wound bed [187].

### 3.3. Cells

**Figure 3:**
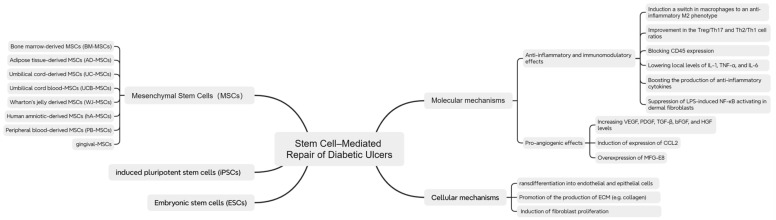
Classification of stem cells in diabetic wounds and their mechanisms involved in cutaneous wound healing.

Stem cells, capable of effectively regulating tissue homeostasis and wound repair, are among the most popular and cutting-edge research directions worldwide. Stem cell-based therapies, particularly mesenchymal stem cell (MSC) therapies, show great potential in diabetic wound repair (Figure 3 and Table 3).

#### 3.3.1. Mesenchymal Stem Cells (MSCs)

MSCs are multipotent stem cells originating from early embryonic development [193]. They can be extracted from various tissues, including bone marrow, adipose tissue, placenta, and umbilical cord [194]. MSCs have the ability to differentiate into various cell types and exert paracrine effects, thus playing an important role in tissue repair [195]. In recent years, the application of MSCs in diabetic wound healing has received significant attention.

MSCs exert effects in diabetic wounds through multiple mechanisms. Vascular damage and subsequent hypoxia are important causes of impaired diabetic wound healing [196]. MSCs have paracrine functions, secreting various pro-angiogenic factors, including VEGF, basic fibroblast growth factor (bFGF), stromal cell-derived factor-1 (SDF-1), keratinocyte growth factor 2 (KGF-2), insulin-like growth factor 1 (IGF-1), placental growth factor (PlGF), and epidermal growth factor (EGF) [197]. These factors promote angiogenesis, improve the vascular microenvironment in the wound area, and ultimately accelerate wound healing [198]. Kerstan et al. found that skin-derived ABCB5 MSCs can activate the HIF-1 pathway in a hypoxic environment, promote VEGF paracrine, and enhance angiogenesis; meanwhile, ABCB5 MSCs differentiate into endothelial lineage cells, directly participating in angiogenesis [188]. MSCs can differentiate into epidermal cells and keratinocytes. Additionally, MSCs can enhance the proliferation and migration of keratinocytes and endothelial cells, promote collagen synthesis, and accelerate wound healing [199].

MSCs also have anti-inflammatory and immunomodulatory effects. MSCs can regulate macrophage phenotypes [200]. Yu et al. showed that adipose tissue-derived mesenchymal stem cells (ADSCs) reduce the expression of pro-inflammatory cytokines, enhance the expression of anti-inflammatory cytokines, decrease the number of classically activated M1 macrophages, and increase the number of alternatively activated M2 macrophages [189]. MSCs can also reduce ROS formation through other mechanisms. Guillén et al. found that MSCs reduce the release of myeloperoxidase and iNOS, and decrease inflammatory cytokines, thereby lowering ROS levels [201]. Furthermore, MSCs can regulate immune function by inhibiting pro-inflammatory T cell differentiation and inducing regulatory T cell accumulation [202]. IL-10 secreted by MSCs plays an important role in inhibiting Th1 and Th17 cells [203]. MSCs overexpressing chemokine receptor 2 (CCR2) can promote Treg accumulation in diabetic wounds [204].

##### Bone Marrow Mesenchymal Stem Cells (BMSCs)

Bone marrow mesenchymal stem cells were the first MSCs discovered and described, typically obtained from bone marrow via bone marrow aspiration. BMSCs have advantages such as high safety and easy availability, with the most mature extraction technology [205]. Multiple studies have shown that BMSCs possess the basic functions of MSCs. Shen et al. demonstrated that neurotrophin-3 (NT-3) stimulation enhances the paracrine function of BMSCs, characterized by increased vasoactive factor secretion. This promotes angiogenesis in diabetic wounds and accelerates diabetic wound healing [190]. Another study showed that BMSCs enhance the production of PDGF and SDF-1, promote collagen deposition, and accelerate wound healing [206].

A meta-analysis showed that BMSCs are more effective than other types of MSCs in diabetes treatment [207]. However, BMSCs face numerous obstacles in clinical applications: they are affected by patient age and physical condition, with their quantity and differentiation potential decreasing with age [208].

##### Adipose-Derived Mesenchymal Stem Cells (ADSCs)

Adipose-derived mesenchymal stem cells are derived from adipose tissue, obtained through liposuction [209]. ADSCs have similar functions to BMSCs. Compared to BMSCs, ADSCs have stronger proliferation and differentiation capabilities [210]. They can be extracted from fat-rich areas such as the abdomen and breasts, with more abundant sources and easier extraction [211]. Additionally, the viability of extracted ADSCs is not affected by age. ADSCs can be transplanted in an allogeneic environment [212]. However, ADSCs are influenced by the extraction site, with variations among donors [213]. Furthermore, studies suggest that ADSCs are more prone to cellular senescence [214].

##### Human Umbilical Cord-Derived Mesenchymal Stem Cells (hUCMSCs)

Similarly to human BMSCs and ADSCs, human umbilical cord-derived mesenchymal stem cells are multipotent. Through multidirectional differentiation and paracrine signaling, hUCMSCs promote diabetic wound repair [215]. HUCMSCs have low immunogenicity and face fewer ethical issues due to their non-invasive collection procedure.

#### 3.3.2. Induced Pluripotent Stem Cells (iPSCs)

Induced pluripotent stem cells are reprogrammed from human cells through ectopic expression of various transcription factors (i.e., Oct4, Sox2, Klf4, and c-Myc (OSKM)), with the morphology, characteristics, and functions of embryonic stem cells (ESCs) and low immunogenicity [216]. iPSCs can be generated from various stem cells [217] and somatic cells [218], with fibroblasts being the earliest and most commonly used cells for iPSC generation [219]. iPSCs can differentiate into all cell types in the skin with low autoimmune responses, thus being considered a promising therapeutic option for diabetic wound healing.

Santarella et al. developed a porous collagen-based scaffold (CS) with ECM derived from iPSC-differentiated fibroblasts, which can improve ECM deposition and enhance angiogenesis in diabetic wound patients [191]. A study on treating diabetic wounds with human iPSC-derived smooth muscle cells (hiPSC-SMCs) demonstrated that hiPSC-SMCs accelerate diabetic wound healing by enhancing the secretion of angiogenic factors and regenerative cytokines to promote cell proliferation and angiogenesis. Additionally, an increased number of M2-type macrophages and a decreased number of M1-type macrophages were observed [192].

Despite encouraging results of cell therapy in diabetic wound treatment, obstacles remain. For example, transplanted cells have low survival rates in the hyperglycemic, inflammatory, and hypoxic wound microenvironment, with reduced migration, differentiation, and paracrine capabilities. Some studies are concerned about the potential risk of tumorigenesis with cell therapy.

### 3.4. Extracellular Vesicles (EVs)

**Figure 4:**
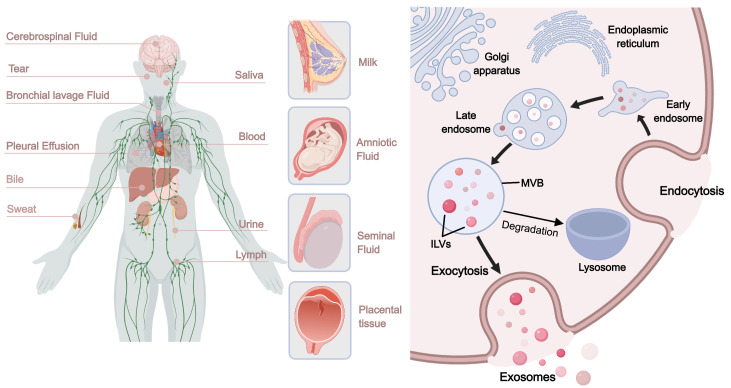
Biogenesis and sources of exosomes. Adapted with permission from [220]. Copyright © 2025, © The Author(s). Published by Oxford University Press.

Extracellular vesicles are heterogeneous lipid bilayer-enclosed structures released by various cell types into the extracellular space, present in almost all physiological fluids, including urine, blood, serum, saliva, bile, lymph, breast milk, cerebrospinal fluid, amniotic fluid, and malignant ascites [220] (Figure 4). In most studies, EVs are generally classified into exosomes (30–150 nm), microvesicles (100–1000 nm), and apoptotic bodies (500–≤5000 nm) based on their origin and size [221]. They carry bioactive molecules such as proteins, lipids, messenger RNA, miRNA, transfer RNA, long non-coding RNA, and mitochondrial DNA, playing a key role in inter-organ and intercellular communication [222]. EVs are currently a research hotspot in biomaterials, with EVs from various sources shown to have positive effects on diabetic wound healing.

In diabetic wound treatment, EVs mainly act as delivery platforms. The inherent structure of EVs (nanoscale size and lipid bilayer) endows them with excellent tissue penetration capabilities [223]. After release, EVs can fuse with target cells through processes such as phagocytosis and endocytosis, releasing their contents to exert effects [224]. Current studies show that in diabetic wound healing, EVs accelerate cell proliferation and migration, promote angiogenesis, inhibit inflammation, and coordinate wound remodeling [225]. These effects are related to their contents, which include various growth factors, cytokines, miRNAs, and DNA, positively promoting tissue repair [226]. Additionally, compared to cell sources, EVs have low immunogenicity, making them ideal for extracellular therapy (Table 4).

#### 3.4.1. Cell-Derived Extracellular Vesicles

##### Bone Marrow Mesenchymal Stem Cell-Derived Extracellular Vesicles (BMSC-EVs)

Bone marrow mesenchymal stem cell-derived extracellular vesicles have received extensive attention due to their low pathogenic microbial infection rate, stable biological properties, low immune rejection rate after transplantation, and high passage number. BMSC-EVs can inhibit inflammation, regulate immunity, enhance angiogenesis and cell migration, thereby accelerating diabetic wound healing [239]. Geng et al. designed a multifunctional hydrogel (designated as MSC-Exos@CEC-DCMC HG) loaded with BMSC-derived exosomes (BMSC-Exos), which is composed of carboxyethyl chitosan (CEC) and dialdehyde carboxymethyl cellulose (DCMC). This hydrogel regulates the wound inflammatory microenvironment in diabetic rats by promoting M2 macrophage polarization, promotes neovascularization, and inhibits bacterial growth, thereby facilitating the healing of chronic diabetic wounds [227]. Furthermore, a study by Qiu et al. demonstrated that BMSC-Exos loaded with miR-221-3p promote angiogenesis and diabetic wound healing by sponging forkhead box P1 (FOXP1), which in turn downregulates Sprouty1 (SPRY1) [240]. Another study demonstrated that BMSCs improve the inflammatory state by reducing the levels of NLRP3 and IL-1β. They also inhibit pyroptosis by suppressing Caspase-1 and decreasing the expression of GSDMD-N, thereby promoting diabetic wound healing [240].

Additionally, BMSC-EVs can regulate cellular autophagy mechanisms. Shi et al. found that BMSC-Exos cultured in a hypoxic environment can deliver miR-4645-5p—which targets and inhibits the expression of MAPKAPK2 to keratinocytes. This activates keratinocyte autophagy, proliferation, and migration, thereby promoting diabetic wound healing in mice [228].

##### Adipose-Derived Stem Cell-Derived Extracellular Vesicles (ADSC-EVs)

Adipose-derived stem cell-derived extracellular vesicles can also inhibit inflammation, regulate immunity, enhance angiogenesis and cell migration, playing an important role in diabetic wound healing. Compared to BMSC-EVs, ADSC-EVs have richer sources and carry a broader range of contents and signaling pathways [241]. Mao et al. showed that ADSC-derived apoptotic bodies (ADSC-ABs) correct the imbalanced M1/M2 macrophage ratio through the miR-20a-5p-mediated janus kinase (JAK)/signal transducer and activator of transcription (STAT) pathway, improve diabetic wound inflammation, and promote angiogenesis, thereby accelerating diabetic wound healing [229]. Chen et al. found that miRNA-146a-modified ADSC-EVs upregulate the expression of serpin family H member 1 (SERPINH1) and phosphorylated extracellular regulated protein kinase (p-ERK), promote fibroblast migration and proliferation, and enhance neovascularization [242]. Wang et al. found that ADSC-EVs accelerate epithelialization and collagen enrichment by inhibiting MMP-9 expression, promoting diabetic wound closure [230].

##### Human Umbilical Cord Mesenchymal Stem Cell-Derived Extracellular Vesicles (hUCMSC-EVs)

Human umbilical cord mesenchymal stem cell-derived extracellular vesicles improve wound healing by promoting angiogenesis, fibroblast proliferation and migration, and regulating inflammatory responses. Teng et al. found that hUCMSC-EVs accelerate diabetic wound healing by promoting M2 macrophage polarization, inhibiting inflammation, and enhancing angiogenesis and collagen deposition [231]. Wei et al. showed that hUCMSC-EVs promote endothelial cell proliferation, migration, and tube formation by delivering miR-17-5p targeting the PTEN/AKT/HIF-1α/VEGF pathway, thereby accelerating diabetic wound healing [232]. Another study demonstrated that human placental mesenchymal stem cells (hPMSCs) combined with platelet-rich plasma (PRP) gel in a gelatin electrospun nanofiber scaffold accelerate wound healing by increasing granulation tissue formation and promoting matrix remodeling [243].

##### Macrophage-Derived Extracellular Vesicles (M-EVs)

Macrophage-derived exosomes play important regulatory roles in diabetic wound healing. Kim et al. found that M2 macrophage-derived exosomes (M2-Exos) directly induce M1-to-M2 macrophage conversion in vitro, improve the M1/M2 macrophage ratio, and alleviate chronic inflammation in diabetic wounds. M2-Exos also carry various cytokines and growth factors that promote wound repair, accelerating wound healing by promoting angiogenesis, re-epithelialization, and collagen deposition [233].

#### 3.4.2. Tissue-Derived Extracellular Vesicles

Compared to cell-derived EVs, tissue-derived EVs offer a more cost-effective and scalable alternative with similar ability to promote diabetic wound healing.

##### Adipose Tissue-Derived Extracellular Vesicles

Compared to MSC-EVs, adipose tissue-derived extracellular vesicles (AT-EVs) have advantages of simple extraction and high yield, emerging as a new and reliable cell-free therapy for clinical diabetic wound treatment. AT-EVs are classified into white adipose tissue-derived EVs (WAT-EVs) and brown adipose tissue-derived EVs (BAT-EVs) based on their origin. Zhang et al. found that in vitro, both WAT-EVs and BAT-EVs accelerate wound healing by promoting wound re-epithelialization, granulation tissue maturation, collagen deposition, and angiogenesis. The study also found that WAT-EVs are more effective than BAT-EVs [244]. Another study showed that AT-EVs can regulate M1-to-M2 macrophage polarization, enhance adipogenesis and angiogenesis, and promote wound healing [234].

##### Plasma-Derived Extracellular Vesicles

Plasma-derived extracellular vesicles also play a role in diabetic wound healing by regulating the local microenvironment. Plasma EVs are mainly derived from platelets [245]. Platelet-rich plasma-derived exosomes (PRP-Exos) have been shown to benefit diabetic wound healing, with immunomodulatory effects and the ability to promote fibroblast proliferation and re-epithelialization. He et al. showed that PRP-Exos accelerate diabetic foot ulcer healing by regulating neutrophil activity, promoting angiogenesis, and promoting macrophage polarization to the M2 phenotype [235]. Wang et al. constructed a hydrogel composed of Pluronic F127 and PRP-Exos (PRP-Exos/Gel). In animal experiments, PRP-Exos/Gel promotes M2 macrophage polarization and inhibits fibroblast ferroptosis, thereby accelerating wound healing [236].

Serum-derived exosomes (Serum-Exos) are another type of plasma-derived exosome. Chen et al. showed that Serum-Exos promote angiogenesis and ECM formation, thereby facilitating wound healing [237]. Another study found that Serum-Exos benefit cell migration and diabetic wound closure both in vivo and in vitro [238].

##### Skin Tissue-Derived Extracellular Vesicles

Skin tissue-derived extracellular vesicles can promote the proliferation and migration of skin cells, accelerating wound healing. Xu et al. demonstrated that extracellular vesicles derived from epidermal stem cells (ESC-EXOs) accelerate diabetic wound healing by inhibiting cellular autophagy and promoting angiogenesis [246]. Wang et al. found that ESC-EXOs promote diabetic wound healing by promoting inflammation resolution, stimulating cell proliferation, enhancing M2 macrophage polarization, and angiogenesis [247].

#### 3.4.3. Biomaterial-Assisted Extracellular Vesicle Delivery Systems

Extracellular vesicles have achieved good results in treating diabetic wounds, but challenges remain [248]. Studies show that EVs are easily rapidly cleared by the immune system, with in vivo half-lives typically ranging from minutes to hours. This short wound retention time greatly limits their application [249].

Biomaterials such as hydrogels and nanofibers can effectively extend the retention time of EVs in wounds, increase their local concentration, and enhance therapeutic effects [250]. Additionally, these biomaterials can achieve sustained support for diabetic wound healing by controlling EV release mechanisms, improving therapeutic efficacy. This approach not only increases EV bioavailability but also effectively reduces wound inflammation [251]. Li et al. designed a GelMA hydrogel-loaded extracellular vesicle derived from keratinocytes, combining EVs from keratinocytes with GelMA hydrogel, which promotes skin microvascular regeneration and wound healing in diabetic mice by activating the PDGF-induced PI3K/AKT pathway [252]. Jv et al. developed an antimicrobial hydrogel based on ε-polylysine and HA to encapsulate EVs. This hydrogel-EVs system increases angiogenesis, enhances cell proliferation, reduces inflammation, and improves tissue structure, accelerating wound healing [253]. Ding et al. prepared a detachable microneedle patch (MN@EVsTβ4) encapsulating thymosin β4 (Tβ4)-modified adipose-derived stem cell vesicles (ADSC-EVs). The study found that MN@EVsTβ4 can slowly release EVTβ4, which promotes diabetic wound healing through the PTEN/PI3K/AKT pathway [254]. Un et al. developed a hydrogel encapsulating PLGA nanoparticles, where the nanoparticles were loaded with disulfiram and wrapped in ADSC-EVs. This hydrogel promotes wound healing by reducing NET formation and facilitating M2 macrophage polarization [255].

### 3.5. Cytokines

Cytokines play a crucial role in diabetic wound healing. In the various biomaterials and delivery strategies mentioned above, regardless of which stage of diabetic wound healing they affect, they ultimately rely on the regulation of growth factors. Thus, delivering cytokines to reshape the diabetic wound microenvironment and enhance tissue regeneration is also a promising strategy.

Growth factors are a class of molecules important in wound healing, mediating, coordinating, and controlling cell interactions, and participating in multiple stages of wound healing [256]. Imbalanced growth factors are also an important factor in delayed diabetic wound healing [257]. Omraninava et al. incorporated bFGF into a collagen hydrogel derived from amniotic membrane (CHA) [258]. The study found that this hydrogel effectively increases VEGF levels, promotes fibroblast proliferation, angiogenesis, and ECM remodeling, reduces inflammatory cell infiltration, and decreases inflammatory cytokine expression, thereby accelerating wound healing.

Interleukins are another class of highly dynamic and complex molecules with important roles, usually interacting through positive or negative regulation and playing key roles in inflammation regulation and tissue repair. Schirmer et al. developed StarPEG-heparin hydrogels, which promote wound healing by improving delayed infiltration of inflammatory cells [259].

At different stages of wound healing, various cytokines exert their effects in an orderly manner, highlighting the spatiotemporal specificity in physiological wound healing. Thus, Li et al. developed a multi-drug hydrogel capable of precisely controlling the sequential release of VEGF-α, silver nanoclusters (AgNCs), and IL-10 [169]. This biomaterial can sequentially participate in critical stages of diabetic wound healing, such as immunoregulation, infection control, and angiogenesis. Emiroglu et al. designed a granular hydrogel capable of isolating and co-carrying IL-6 and VEGF [260]. These two novel biomaterials represent a new approach and trend. In the future, novel biomaterials with spatiotemporally specific delivery and isolation capabilities, capable of acting on multiple stages of diabetic wound healing, will further promote diabetic wound healing.

## 4. Challenges and Future Perspectives

Research and application of diabetic wound healing face numerous challenges. Firstly, the wound healing process in diabetic patients is complex, influenced by multiple factors such as blood glucose control, microcirculatory disorders, and immune dysfunction [261]. Secondly, existing treatments often fail to effectively address chronic wound healing, highlighting the urgent need to develop new biomaterials and therapeutic methods [262]. Future research should focus on improving diabetic wound healing outcomes through innovative biomaterial-based regulatory therapies.

### 4.1. Development Trends of Multifunctional Intelligent Biomaterials

With advancements in biomaterials science, the development of multifunctional intelligent biomaterials has become an important trend in diabetic wound treatment. Such materials not only have good biocompatibility but also can respond to changes in the wound microenvironment to achieve precise treatment [263]. For example, responsive materials can release anti-inflammatory drugs or growth factors in response to changes in wound pH, temperature, or glucose concentration, promoting healing [264]. Zhang et al. developed a pH-responsive injectable sodium alginate/carboxymethyl chitosan hydrogel [265]. This hydrogel has excellent ECM-like properties in irregular wound shapes and responds to acidic microenvironments by releasing agents that promote cell migration, alleviate oxidative stress, and reduce mitochondrial damage.

Additionally, the application of multi-stimulus synergistic response technology enables biomaterials to exert multiple functions under different stimuli, such as antibacterial effects, promotion of angiogenesis, and acceleration of cell migration, greatly improving wound healing efficiency [266]. Li et al. designed a glucose and pH dual-responsive hydrogel, which shows good responsiveness to acidic conditions and high glucose levels, with strong antibacterial activity and antioxidant capacity [267]. Multifunctional biomaterials that can act on different aspects of diabetic wound healing at different stages are also a new direction in biomaterial development [268]. Zhang et al. prepared a lipopeptide/Ti3C2Tx MXene nano-hybrid. Ti3C2Tx has antibacterial, antioxidant, and pro-angiogenic capabilities, and drug-free lipopeptides also exhibit antibacterial activity. Thus, this hybrid can effectively heal bacteria-infected diabetic wounds [269]. Zhong et al. reported a novel immunosuppressive pure DNA hydrogel (Is-pDNAgel) [270]. At the molecular level, its high-density negative charge and immunosuppressive domains work together to effectively clear free chemokines, inhibit multiple pro-inflammatory signaling pathways, and enhance anti-inflammatory efficacy. At the cellular level, it can inhibit macrophage and neutrophil infiltration, promote migration and proliferation of endogenous endothelial cells, enhance re-epithelialization and neovascularization, and accelerate wound healing.

### 4.2. Key Issues in Clinical Translation

Although multifunctional intelligent biomaterials show good results in laboratory studies, their clinical translation faces numerous challenges. Firstly, long-term safety and efficacy evaluations are critical to ensure successful clinical application of new materials [271]. Long-term animal experiments are required to verify the toxicity, immunogenicity, and carcinogenicity of biomaterials and their degradation products; for example, stem cells pose potential carcinogenic risks [272].

Higher differentiation potential of stem cells leads to a greater likelihood of uncontrolled proliferation, i.e., stronger tumorigenic risk. During long-term in vitro culture, cells may accumulate chromosomal abnormalities (e.g., telomere shortening and gene mutations), thereby triggering “abnormal proliferation.” Donor-specific factors (e.g., age and health status) cause significant variations in the core functions (e.g., viability, differentiation capacity, and immunogenicity) of stem cells from different donors. Cell manufacturing processes further exacerbate this donor variability. Collectively, these factors result in inconsistent efficacy of cell therapies and increase the difficulty of clinical research.

Furthermore, differences in regulatory strategies for stem cell therapies across countries/regions create barriers to cross-border research and clinical applications. Under the U.S. Food and Drug Administration (FDA) framework, stem cells are classified as biological products. Stem cells used for homologous purposes, not combined with other substances, minimally manipulated, and without systemic effects or reliance on metabolic activity may be exempt from FDA premarket approval. However, such stem cells must still comply with the safety standards, donor eligibility requirements, and manufacturing specifications detailed in Good Manufacturing Practice (GMP). Donors must also undergo screening for infectious pathogens, such as hepatitis B virus (HBV) and human immunodeficiency virus (HIV). Stem cell therapies that do not meet these standards require submission of multiple applications to obtain marketing and distribution approval. In Europe, stem cells are categorized into “low-risk cell products” (e.g., local injection of autologous MSCs) and “high-risk cell products” (e.g., systemic infusion of iPSC-derived cells). Low-risk products are eligible for a streamlined approval process. However, inconsistent implementation of regulatory standards among EU member states increases the difficulty of coordinating cross-border multicenter clinical trials.

Furthermore, most research on biomaterials is conducted using rodent models [273]. Rodent studies serve as a critical starting point for exploring the basic mechanisms of exosomes, with advantages of low cost, minimal ethical restrictions, and high experimental controllability. However, compared to human skin, differences exist in the relative thickness of the epidermis and dermis, epidermal turnover time, and skin-resident immune cells. Such species-specific differences weaken the guiding significance of experimental conclusions for human applications. Therefore, in vitro experiments with human primary cells and non-human primate validation should be supplemented before clinical trials. Additionally, unified animal models and clinical evaluation criteria must be established, including wound closure rate, angiogenesis indicators, dynamic microbial load monitoring, and cell count, to improve the comparability of cross-study results [274].

Secondly, large-scale production and cost control are important factors affecting the promotion of new materials. Long-term stability requires biomaterials to maintain structural integrity and functional durability in complex wound microenvironments while resisting premature degradation caused by enzymatic hydrolysis, hydrolysis, or mechanical stress [275]. Strategies such as advanced cross-linking (e.g., dynamic covalent bonds and nanocomposite reinforcement) or enzyme-resistant polymers (e.g., PEGylation) can improve stability, but the synthesis difficulty and biocompatibility must be balanced [276].

How to reduce production costs while ensuring material performance is a key issue for future research. Technologies such as DNA nanomaterials and stem cell culture are costly, requiring optimized preparation processes and exploration of alternative biodegradable polymer materials [277]. Large-scale purification and quality control of bioactive components such as exosomes and cytokines are industrialization bottlenecks, requiring the development of efficient, low-cost separation technologies [278]. For example, MSCs derived from adult tissues have limited proliferation capacity, resulting in low yields of EVs. MSCs derived from pluripotent stem cells offer a viable solution due to their higher proliferation rate and ability to produce large amounts of EVs, but obstacles remain, including the need for standardized protocols for EV generation, isolation, and characterization [220]. Additionally, clinical trial design and implementation need to be more rigorous to ensure the efficacy and safety of new materials in real patients.

Since exosome preparation relies on cell populations, information about these cell populations must be registered for traceability, including key details such as cell type and tissue origin. Donors must meet strict exclusion criteria, e.g., confirmation of no infection with pathogens like HIV or HBV. Parameters related to the purity, sterility, potency, and characteristics of exosome production batches must be verified through testing. This process may require the International Society for Extracellular Vesicles (ISEV) or national regulatory authorities to establish unified basic standards. Among these steps, batch quality control is particularly critical, requiring quantitative testing of exosome impurity content, homogeneity, sterility, and toxicological effects. Furthermore, the design and implementation of clinical trials need further refinement to fully verify the efficacy and safety of novel materials in real patient populations.

### 4.3. Personalized Therapy and Multidisciplinary Integration

Personalized therapy is an important direction in diabetic wound healing research. Customized material design based on patient wound characteristics can improve treatment targeting and efficacy. Integrating biomedical engineering, materials science, and clinical medicine enables precise grasp of individual patient differences, thereby formulating more effective treatment plans [263]. Different biomaterials should be designed for different types of diabetic wounds to meet their specific biological needs. Based on individual characteristics such as wound microbial flora and inflammatory factor profiles, customized biomaterials can be designed.

Currently, a growing number of new materials targeting different sites of diabetic wounds have been developed. For example, cerium oxide (CeO_2_) nanozymes possess a unique redox-active surface. Zwitterionic materials can effectively reduce bacterial adhesion and prevent biofilm formation. Natural polyphenols such as resveratrol exhibit antioxidant activity and inhibit the formation of AGEs. Different types of materials can be combined based on the pathological characteristics of patients’ skin lesions to achieve specific therapeutic effects.

**Figure 5:**
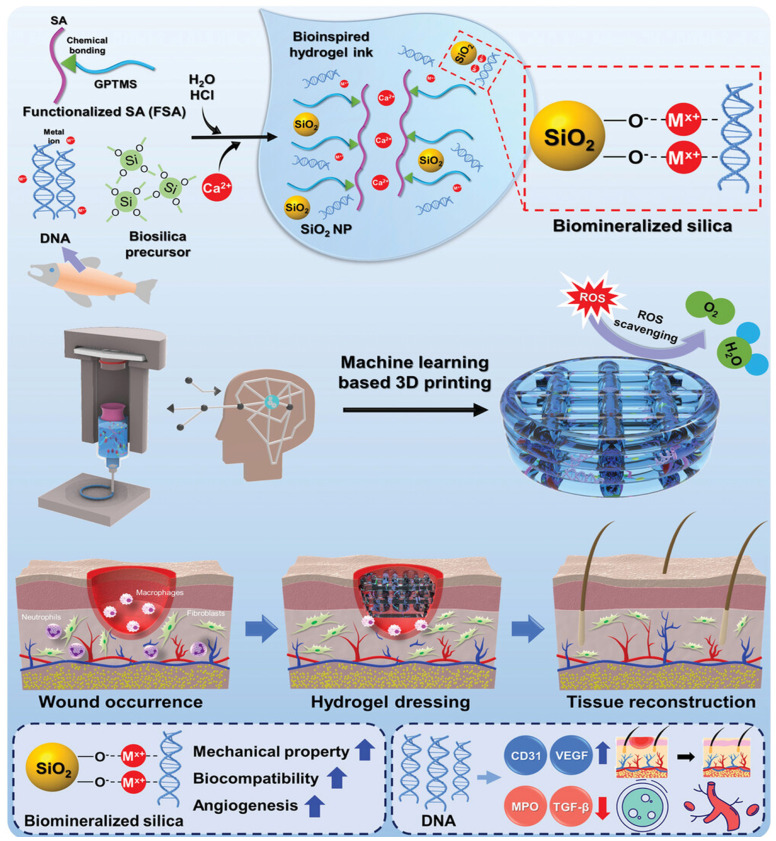
Schematic diagram of the fabrication process of bioinspired 3D-printed hydrogels using DNA-induced biomineralization. Adapted with permission from [281]. © 2023 The Authors. Advanced Science published by Wiley-VCH GmbH.

The rapid development of new nanotechnologies (e.g., photothermal therapy (PTT), layer-by-layer (LBL) self-assembly, and 3D printing) has brought hope to this concept [279]. For example, 3D-printed scaffolds for wound dressings have multiple advantages, such as adjustable size characteristics (e.g., area, thickness, or pore size), easy drug loading, and flexible material use [280]. Using 3D printing technology, a naturally bioactive wound dressing based on biomineralized silica NPs and salmon sperm natural DNA embedded in functionalized alginate (FSA) hydrogel has been developed for acute and chronic wound treatment [281] (Figure 5).

Furthermore, multidisciplinary collaboration will promote the research, development, and application of new technologies and materials, driving overall progress in the field of diabetic wound healing. Cross-disciplinary cooperation among materials scientists, biologists, and clinicians is crucial, requiring the establishment of a full-chain translation system from material design and mechanism research to clinical trials.

## 5. Conclusions

Various biomaterials like DNA nanomaterials, peptide hydrogels, cells and exosomes have advantages over traditional treatments in angiogenesis, inflammation regulation, antibacterial activity and cell function promotion for wound healing. However, obstacles in the clinical translation of biomaterials cannot be ignored. Issues such as cell survival, immune response and large-scale production need to urgently be solved. The preclinical efficacy proven in mouse models awaits verification by randomized clinical trials. These challenges require in-depth exploration and research by researchers in future work. In diabetes wound research, attention should be paid not only to biomaterials’ impact on diabetes wound healing, but also on blood glucose levels, the “ultimate goal” of diabetes treatment. Therefore, combined therapies via multiple approaches and targets are better options. Of course, this requires more consideration and research on their temporal and spatial order. Certainly, given difficulties like immune response and large-scale production, personalized targeted therapy may be another good choice.

## Figures and Tables

**Table 1:** DNA-based Biomaterials Used in Experimental Studies.

Biomaterials	Model	Results	Refs
TDNs Society	Diabetic Wistar rat full-thickness cutaneous wound	Accelerating vascularization, epithelialization, collagen deposition, and collagen alignment	[146]
TDNs	Diabetic mice (db/db) full-thickness cutaneous wound	Regeneration of the epidermis, capillaries, and collagen	[147]
TDN loaded with asPNAs	MRSA	Antibacterial activity	[148]
TDN loading Ampicillin	MRSA	Antibacterial activity	[149]
TDN loaded with GL13K	Pseudomonas gingivalis	Antibacterial activity	[150]
ASOs-tFNAs	*S. mutans* UA159	Antibacterial activity	[151]
p@tFNA	Diabetic mice (db/db) full-thickness cutaneous wound	Promoting angiogenesis and antioxidant activity	[152]
tFNA-Apt02-DMOG	Diabetic mice (Balb/c) full-thickness cutaneous wound	Promoting angiogenesis	[153]
L12 loaded DNA hydrogels	Healthy mice (C57BL/6J) full-thickness cutaneous wound	Antibacterial activity	[154]
VEGF-GAHCM	Diabetes mice (C57BL/6J) full-thickness cutaneous wound	Promoting angiogenesis and collagen deposition	[155]
PEI/PDRN@SA/SCS hydrogels	Kunming mice full-thickness cutaneous wound	Promoting cell proliferation and Antibacterial activity	[156]

**Table 2:** Peptide hydrogel Used in Experimental Studies.

Biomaterials	Model	Results	Refs
Fmoc-Phenylalanine Nanofibrillar Hydrogel	*S. aureus* and *E. coli*	Antibacterial activity	[174]
L9-Ag Hydrogel	*E. coli* (ATCC 25922)	Antibacterial activity	[175]
Antimicrobial Peptide-Functionalized Mesoporous Hydrogels	Diabetic rat (SD) full-thickness cutaneous wound	Antibacterial activity	[176]
Peptide loaded self-healing hydrogel	Diabetic rat (SD) full-thickness cutaneous wound	Enhancing M1-to-M2 macrophage polarization, promoting asngiogenesis, and collagen deposition	[177]
HBD-2 COL-CS scaffold	Diabetic rat (Wistar) full-thickness cutaneous wound	Accelerating cell migration and angiogenesis; anti-inflammatory and antibacterial	[178]
PGF@ALG/PLGA hydrogel	Diabetic mice (db/db) full-thickness cutaneous wound	Enhancing M1-to-M2 macrophage polarization; Antibacterial activity	[179]

**Table 3:** Cell Used in Experimental Studies of Diabetic Wound Healing.

Cell Source	Model	Results	Refs
ABCB5 MSC	Diabetic foot patients	Promoting angiogenesis	[188]
ADSC	Diabetic rat (ZDF) full-thickness cutaneous wound	Enhancing M1-to-M2 macrophage polarization	[189]
BMSC	Diabetes mice (C57BL/6J) full-thickness cutaneous wound	Promoting angiogenesis and cell migration	[190]
iPSC	DFU patient-matched fibroblasts	Promoting angiogenesis and ECM deposition	[191]
hiPSC-SMC	Diabetic mice full-thickness cutaneous wound	Promoting angiogenesis	[192]

**Table 4:** Extracellular Vesicles Used in Experimental Studies of Diabetic Wound Healing.

EVs Source	Model	Results	Refs
BMSC-EVs	Diabetic rat (SD) full-thickness cutaneous wound	Enhancing M1-to-M2 macrophage polarization; promoting angiogenesis	[227]
BMSC-EVs	Diabetic mice (db/db) full-thickness cutaneous wound	Induce keratinocyte autophagy; activate keratinocyte proliferation and migration	[228]
ADSC-ABs	Diabetic rat full-thickness cutaneous wound	Enhancing M1-to-M2 macrophage polarization; promoting angiogenesis	[229]
ADSC-EVs	Diabetic mice full-thickness cutaneous wound	Improve re-epithelialization and collagen; inhibit the expression of MMP-9	[230]
hUCMSCs-EVs	Diabetic rat (SD) full-thickness cutaneous wound	Enhancing M1-to-M2 macrophage polarization; promoting angiogenesis; improve re-epithelialization and collagen	[231]
hUCMSCs-EVs	Diabetic mice (db/db) full-thickness cutaneous wound	Promoting angiogenesis	[232]
M2-EVs	Diabetic mice (Balb/c) full-thickness cutaneous wound	Enhancing M1-to-M2 macrophage polarization; promoting angiogenesis	[233]
AT-EVs	Diabetic rat (SD) full-thickness cutaneous wound	Enhancing M1-to-M2 macrophage polarization; promoting Adipose tissue regeneration	[234]
PRP-EVs	Diabetic mice (Balb/c) full-thickness cutaneous wound	Enhancing M1-to-M2 macrophage polarization; promoting angiogenesis	[235]
PRP-EVs	Diabetic mice (db/db) full-thickness cutaneous wound	Inhibit fibroblast ferroptosis; enhancing M1-to-M2 macrophage polarization	[236]
Serum-EVs	Diabetic mice full-thickness cutaneous wound	Promote angiogenesis and ECM formation	[237]
EpiSC-EVs	Diabetic mice (db/db) full-thickness cutaneous wound	Promote angiogenesis	[238]

## Data Availability

No new data were created or analyzed in this study.
